# Right ventricular functional analysis utilizing first pass radionuclide angiography for pre-operative ventricular assist device planning: a multi-modality comparison

**DOI:** 10.1186/s13019-017-0652-y

**Published:** 2017-10-10

**Authors:** Ryan Avery, Kevin Day, Clinton Jokerst, Toshinobu Kazui, Elizabeth Krupinski, Zain Khalpey

**Affiliations:** 1 Department of Medical Imaging, Banner - University Medical Center, 1501 N. Campbell Ave, PO Box 245067, Tucson, AZ 85724 USA; 2 Department of Radiology, Mayo Clinic Hospital – Phoenix, Phoenix, AZ USA; 3 Department of Surgery, Division of Cardiothoracic Surgery, Banner – University Medical Center, Tucson, AZ USA; 4 Department of Radiology and Imaging Science, Emory University Hospital, Atlanta, US Georgia

**Keywords:** Magnetic resonance imaging, First pass radionuclide angiography, Multigated acquisition radionuclide angiography, Automatic implantable cardiac device, Right ventricular ejection fraction, Left ventricular ejection fraction

## Abstract

**Background:**

Advanced heart failure treated with a left ventricular assist device is associated with a higher risk of right heart failure. Many advanced heart failures patients are treated with an ICD, a relative contraindication to MRI, prior to assist device placement. Given this limitation, left and right ventricular function for patients with an ICD is calculated using radionuclide angiography utilizing planar multigated acquisition (MUGA) and first pass radionuclide angiography (FPRNA), respectively. Given the availability of MRI protocols that can accommodate patients with ICDs, we have correlated the findings of ventricular functional analysis using radionuclide angiography to cardiac MRI, the reference standard for ventricle function calculation, to directly correlate calculated ejection fractions between these modalities, and to also assess agreement between available echocardiographic and hemodynamic parameters of right ventricular function.

**Methods:**

A retrospective review from January 2012 through May 2014 was performed to identify advanced heart failure patients who underwent both cardiac MRI and radionuclide angiography for ventricular functional analysis. Nine heart failure patients (8 men, 1 woman; mean age of 57.0 years) were identified. The average time between the cardiac MRI and radionuclide angiography exams was 38.9 days (range: 1 - 119 days). All patients undergoing cardiac MRI were scanned using an institutionally approved protocol for ICD with no device-related complications identified. A retrospective chart review of each patient for cardiomyopathy diagnosis, clinical follow-up, and echocardiogram and right heart catheterization performed during evaluation was also performed.

**Results:**

The 9 patients demonstrated a mean left ventricular ejection fraction (LVEF) using cardiac MRI of 20.7% (12 – 40%). Mean LVEF using MUGA was 22.6% (12 – 49%). The mean right ventricular ejection fraction (RVEF) utilizing cardiac MRI was 28.3% (16 – 43%), and the mean RVEF calculated by FPRNA was 32.6% (9 – 56%). The mean discrepancy for LVEF between cardiac MRI and MUGA was 4.1% (0 – 9%), and correlation of calculated LVEF using cardiac MRI and MUGA demonstrated an R of 0.9. The mean discrepancy for RVEF between cardiac MRI and FPRNA was 12.0% (range: 2 – 24%) with a moderate correlation (*R* = 0.5). The increased discrepancies for RV analysis were statistically significant using an unpaired t-test (*t* = 3.19, *p* = 0.0061). Echocardiogram parameters of RV function, including TAPSE and FAC, were for available for all 9 patients and agreement with cardiac MRI demonstrated a kappa statistic for TAPSE of 0.39 (95% CI of 0.06 – 0.72) and for FAC of 0.64 (95% of 0.21 – 1.00).

**Conclusion:**

Heart failure patients are increasingly requiring left ventricular assist device placement; however, definitive evaluation of biventricular function is required due to the increased mortality rate associated with right heart failure after assist device placement. Our results suggest that FPRNA only has a moderate correlation with reference standard RVEFs calculated using cardiac MRI, which was similar to calculated agreements between cardiac MRI and echocardiographic parameters of right ventricular function. Given the need for identification of patients at risk for right heart failure, further studies are warranted to determine a more accurate estimate of RVEF for heart failure patients during pre-operative ventricular assist device planning.

## Background

Advanced left heart failure therapy is focused on hospitalization and heart transplantation given the poor outcomes of medical therapy alone in these patients [1, 2]. However, the finite supply of donor hearts has limited transplantation to 4500 annual cases worldwide with 2200 of these performed in the United States [3]. The introduction of the left ventricular assist device (LVAD) has become a valuable tool providing a bridge to transplant till a donor organ becomes available, or a bridge to therapy with evidence suggesting an increased 1-year survival compared to medical management [3–6]. For patients who are not a transplant candidate, LVAD implantation has also been approved as a destination therapy offering both improved mortality rate and quality of life [7, 8].

Given the prevalence of advanced heart failure, potential LVAD recipients in the US is estimated to be 150,000 to 250,000 patients [3, 9]. Further studies have noted that despite the improvements in 1-year survival rates, LVADs continue to have significant morbidity and mortality related to development of right ventricular failure (RVF) after LVAD placement hypothesized to be related to the LVAD providing only left ventricular support [10, 11]. While the clinical decision regarding the selection of univentricular or biventricular support device is multifactorial, the risk of post-device placement RVF warrants that the size and function of the right ventricle (RV) should be an integral component in this decision-making process [12–14]. Furthermore, RV function in heart failure patients correlates with mortality risk, and is more predictive of survival than exercise testing and left ventricular ejection fraction (LVEF) [15–18]. If right ventricular function is compromised, the risk of post-procedural RVF is high, and LVAD implantation into patients needing biventricular support will result in an increased risk of right ventricular failure and poor clinical outcomes [11, 19–23].

While echocardiography is a widely available and non-invasive method of ventricular functional analysis with RV function assessed by both tricuspid annular plane systolic excursion (TAPSE) and fractional area of change (FAC), monitoring RV function and size with ultrasound parameters is not currently advocated for definitive RV evaluation [18]. Radionuclide angiography was determined to be the first, noninvasive, quantitative assessment of ventricular function [24], and more recently cardiac MRI cinematic images of the ventricles provides a level of accuracy allowing it be considered the “gold standard” for calculation of both LVEF and RVEF and ventricular volumetric parameters [25, 26].

Despite the advantages of cardiac MRI, ventricular function using radionuclide angiography continues to be favored with the multi-gated acquisition technique (MUGA) used to calculate LVEF, and the first-pass radionuclide angiography (FPRNA) technique used to calculate the RVEF. Multiple factors contribute to the continued preferred status of radionuclide angiography in societal guidelines; particularly, the limited availability of cardiac MRI, and that is contraindicated in patients with implantable cardiac devices (ICDs) [27–33].

In a continued effort for accurate assessment of both left and right ventricular function for patients prior to LVAD placement, a workflow was implemented at our institution for patients with conventional ICDs allowing for scanning with a 1.5 T MRI utilizing a published evidence-supported protocol [34–40]. Our goal was to accurately evaluate LV and RV function prior to LVAD placement by comparing the performance of radionuclide angiography to the reference standard values of cardiac MRI and other available hemodynamic and echocardiographic parameters of right ventricular functional analysis with the objective of preventing both post-LVAD implantation RVF and its associated mortality risk.

## Methods

### Study design

An IRB-approved, retrospective review from January 2012 through May 2014 was performed at a single US academic medical center. The review identified all adult advanced heart failure patients treated with an ICD who were being evaluated for either LVAD placement or heart transplantation. All patients were included who underwent left and right ventricular function evaluation with both cardiac MRI and radionuclide angiography within 120 days. A comparison of the calculated left and right ventricular ejection functions was performed comparing cardiac MRI with radionuclide angiography. The electronic medical record for each patient who met inclusion criteria was reviewed for the clinical course, specifically whether the patient went on to receive a LVAD. Medical record review also included performance of any echocardiogram or right heart catheterization performed during the evaluation period. While these exams do not provide a calculated ejection fraction, that right ventricular functional parameters from these exams have been shown to be predictive of post-operative right ventricular failure after LVAD placement [41].

Cardiac MRI exams included in the study required the performance of a complete short-axis series of the ventricles using cine EKG-gated sequences. Radionuclide angiography examinations included in the study required performance of both a MUGA and FPRNA exam for respective LVEF and RVEF evaluation. Right ventricular functional parameters from available echocardiograms included tricuspid annular plane systolic excursion (TAPSE) and fractional area of change (FAC), and from available right heart catheterization (RHC) parameters included central venous pressure (CVP), mean pulmonary artery pressure (MPAP), and right ventricular stroke work index (RVSWI) utilizing thermodilution.

### Study population

Nine patients including 8 (89%) men and 1 (11%) woman with a mean age of the patients was 57.0 years (median: 60.0, range: 28 - 66) underwent ventricular ejection fraction evaluation with both cardiac MRI and radionuclide angiography for pre-operative heart failure evaluation for planning of LVAD placement or heart transplantation. Four patients were diagnosed with ischemic cardiomyopathy, and 5 patients were diagnosed with nonischemic cardiomyopathy. The average time interval between the cardiac MRI and radionuclide angiography (MUGA and FPRNA) was 38.9 days (range: 1 - 119 days). The average dose of intravenously administered technetium-99 m labeled RBCs for radionuclide angiography was 23.0 mCi (range 20.1 – 25.0 mCi).

All patient had a transthoracic echocardiogram performed during their evaluation with the average time between echocardiogram and cardiac MRI of 41 days (range: 1 – 125 days). Additionally, 7 of the 9 patients underwent RHC with an average time between RHC and cardiac MRI of 47 days (range 1-125 days).

All patients evaluated had previously undergone ICD placement prior to cardiac MRI examination, which was performed using an institutionally approved ICD safe protocol. There were no device-related complications from cardiac MRI in our study population.

### Cardiac magnetic resonance examination acquisition and analysis

All patients were screened by a multi-specialty team prior to performance of the MRI. Screening required a cardiology evaluation consisting of electrophysiology laboratory interrogation of the ICD. The approved selection criteria required all cardiac leads in place for more than 6 weeks; stable pace, sense, and impedance parameters, normal battery function, and a stable non-paced heart rhythm with a heart rate greater than 50 beats per minute. If any of parameters were unable to be achieved, the patient was unable to undergo an MRI.

Prior to the MRI examination, the ICD was programmed to a sensing-only mode without cardiac pacing within the EP laboratory by electrophysiology-specialized cardiologists. Ventricular tachycardia and ventricular fibrillation detection, a feature in implantable defibrillators, was also turned off if applicable. The patient was monitored during the entire exam, and after the MRI was performed the patient was immediately returned to the electrophysiology laboratory where the ICD was reprogrammed to its original settings. Performance of the Cardiac MRI was done under the supervision of a dedicated advanced cardiac imaging radiologist and an MRI physicist who were both present for the entirety of each examination.

Each cardiac MRI was performed using a 1.5 T Siemens Magnetom Aera (Siemens Healthcare, Erlangen, Germany) with a multiphase array body coil. Specific absorption rate was limited to 1.5 W/kg for a maximum of 30 min. Gradient recalled echo (GRE) images were obtained for all bright blood and cine gated images to minimize susceptibility artifact related to the ICD battery pack. All images were obtained during breath hold. GRE cine gated images of the LV long-axis were performed in the 2, 3, and 4 chamber orientations for ventricular assessment. For complete ventricular analysis, GRE cine gated short axis series including complete coverage both ventricles were performed. The short axis series consisted of a stack of 8 mm thick images with a 10% distance factor between images. No gadolinium-based contrast agents were administered during the examination.

Cardiac MRI interpretation and offline post-processing analysis (CVI42, Circle Cardiovascular Imaging, Calgary, Canada) of ventricular ejection fractions was performed by consensus between two fellowship trained cardiovascular radiologists. Ventricular ejection fraction calculations were performed by manual contouring of the endocardial border of both ventricles during both end-diastole and end-systole utilizing all relevant sequences of the short axis series. Offline post-processing analysis also calculated end diastolic, end systolic, and stroke volumes of both ventricles. The results of the original studies were collected retrospectively, and were not reinterpreted.

### Multi gated acquisition scan acquisition and analysis

Ventricular ejection fraction determined by radionuclide angiography was performed using an intravenous injection of Technetium-99 m-tagged red blood cells (RBC). Radiopharmaceutical tagging was performed using an in vitro labelling technique to ensure a high percentage of RBC tagging, and to prevent the injection of free Technetium-99 m. The RBCs for tagging were acquired from a 1 - 3 mL intravenous blood sample from the patient, which was subsequently tagged with radiopharmaceutical by a commercial tagging kit (UltraTag™, Mallinckrodt Pharmaceuticals, Maryland Heights, MO) prior to reinjection. After tagged RBC reinjection, the FPRNA examination was performed by obtaining dynamic nuclear angiography images in the RAO 30° projection at a rate of 50 msec/frame for a total acquisition of 440 frames. Additional, gated planar images in the RAO 30° projection were subsequently obtained for 300 s. After the FPRNA examination was performed, the MUGA portion of the radionuclide angiography examination was performed for LVEF evaluation. The MUGA images were acquired with gated images performed in the LAO 45° projection for 10 min.

**Fig. 1:**
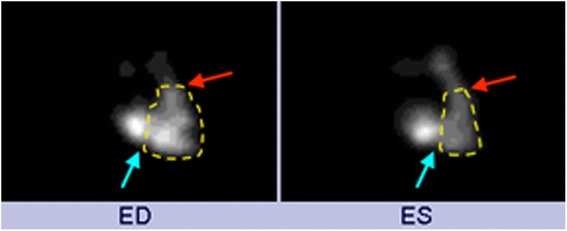
Images of end diastole (ED) and end systole (ES) from a first pass radionuclide angiography. Images were obtained in the 30° RAO position, and computer contouring of the right ventricle was manually performed (yellow dashed line). Attention to including the right ventricular outlow tract to the pulmonary valve (red arrow) and base of the right ventricle to the right atrium (blue arrow) was performed

RVEF was calculated by performance of computer contouring of the right ventricle in the RAO 30° position throughout the cardiac cycle and computer detection of the end diastole (ED) and end systole (ES) frames. Right ventricular contouring was performed by identification of the tagged RBC within the RV cavity and contouring of the endocardial border of the RV while excluding the right atrium and main pulmonary artery (Fig. 1). LVEF was calculated by the MUGA portion of the examination that was performed in a similar fashion by contouring the endocardial border of the LV throughout the cardiac cycle in either the LAD 45° or 70° projection given the anatomic position of the heart, and computer detection of ED and ES. All contouring was performed an experience nuclear technologist, and two fellowship trained nuclear medicine radiologists confirmed exam quality and interpreted the FPRNA and MUGA images. The results were obtained retrospectively and were not reinterpreted.

### Echocardiographic acquisition and analysis

All patients underwent a comprehensive 2D transthoracic echocardiographic examination performed during rest from the left parasternal, apical and subcostal windows using the GE ultrasound (Chicago, IL). RV assessment included TAPSE, which was measured by M-mode to assess RV longitudinal function by tracing movement of the tricuspid valve annulus from end diastole to end systole. RV area was measured by planimetry of the RV cavity in the apical view along the RV free wall to the apex and back to the tricuspid annulus for both systole and diastole to obtain the end-systolic area (ESA) and end-diastolic areas (EDA) respectively. Fractional area change (FAC) was calculated as [(EDA-ESA)/EDA] × 100.

### Invasive hemodynamic measurements

RHC was performed through the right internal jugular or femoral approach in 7 subjects at rest using the Swan-Ganz pulmonary artery catheter in the supine position. Direct measurements of RAP, pulmonary artery systolic pressure, PA diastolic pressure (PADP), mean PA pressure (MPAP), cardiac output (CO) by thermodilution, cardiac index (CI) and pulmonary artery wedge pressure (PAWP) were obtained and real-time tracings were displayed on a monitor. Right ventricular stroke work index (RVSWI) was calculated as (CI/HR) x MPAP × 0.0144.

## Results

Statistical analysis ventricular ejection fractions of the nine patients who met inclusion criteria included calculation of discrepancies and correlation coefficient between the ejection fractions provided by cardiac MRI and radionuclide angiography, which provided LVEF by MUGA and RVEF by FPRNA. Discrepancy values were evaluated for statistical significance using an unpaired t-test.

The mean RVEF using cardiac MRI was 28.3% (median 27.0%, range: 16 – 43%). The mean RVEF calculated using FPRNA was 32.6% (median 32.0%, range: 9 – 56%). The mean LVEF calculated using cardiac MRI was 20.7% (median 21.0%, range: 12 – 40%), and mean LVEF calculated using MUGA was 22.6% (median 19.0%, range: 12 – 49%).

**Fig. 2:**
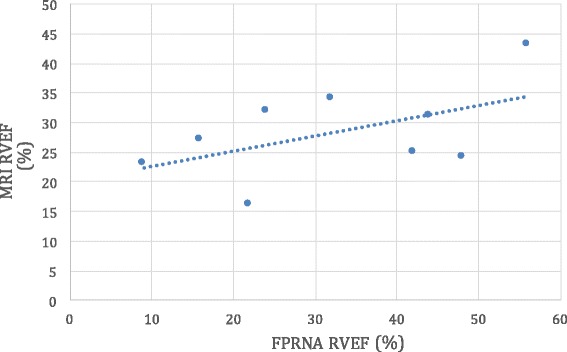
Correlation of MRI derived right ventricular ejection fraction (MRI RVEF) to radionuclide derived right ventricular ejection fraction utilizing a first-pass radionuclide angiography (MUGA RVEF). A moderate correlation (*R* = 0.5) was determined when comparing RVEF calculated by both modalities

**Fig. 3:**
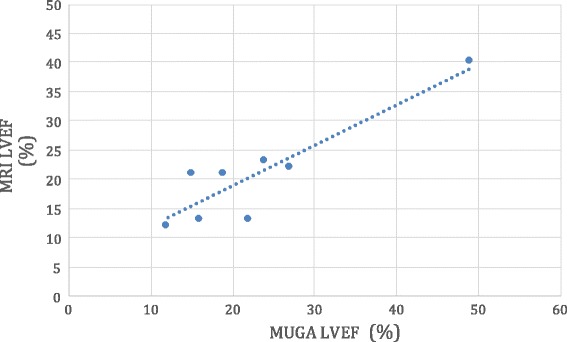
Correlation of MRI derived left ventricular ejection fraction (MRI LVEF) to radionuclide derived left ventricular ejection fraction utilizing a multi-gated acquisition (MUGA LVEF). Correlation between the 2 modalities demonstrates a strong correlation (*R* = 0.9)

The mean RVEF discrepancy between cardiac MRI and FPRNA was 12.0% (median 13.0%, range: 2 – 24%). The mean LVEF discrepancy between cardiac MRI and MUGA was 4.1% (median 3.0%, range: 0 – 9%). The difference in discrepancies between the two modalities for RV and LV analysis was statistically significant using an unpaired t-test (*t* = 3.19, *p* = 0.006) (Table 1). The correlation coefficient between FPRNA generated RVEF and cardiac MRI RVEF was 0.5 (Fig. 2). The correlation coefficient between LVEF calculated using MUGA and cardiac MRI was 0.9 (Fig. 3).

Agreement of ventricular function using reference standard cardiac MRI to echocardiogram was performed for all 9 patients by performed by calculation of a Kappa statistic comparing mild, moderate, and severe RV dysfunction for both TAPSE and FAC to mild, moderate, and severe RV dysfunction determined by calculated RVEFs determined by cardiac MRI [42, 43] (Table 2). The kappa statistic for RV function agreement between echocardiogram TAPSE agreement and cardiac MRI was 0.39 with a 95% CI of 0.06 - 0.72. The kappa statistic between echocardiogram FAC and Cardiac MRI showed a higher agreement of 0.64 with a 95% CI of 0.21 - 1.00. RHC parameter comparison demonstrated abnormal CVP, MPAP, and RVSWI in 6 of the 7 patients who had underwent RHC during heart failure evaluation (Table 3).

Of the nine patients in this study, three patients subsequently received an LVAD (HeartMate II Ventricular Assist Device, Thoratec Corporation, Pleasanton, California) for left ventricular support. One of the patients who underwent LVAD implantation was successfully bridged to transplant while the other two are still awaiting transplant. One patient identified for LVAD placement did not receive their LVAD secondary to complications from ischemic bowel disease that resulted in the patient expiring prior to LVAD placement. One patient identified for LVAD placement was deemed unsuitable for transplant due to lack of social support and inability to comply with smoking cessation program. Three patients improved clinically on optimal medical therapy and optimized medical management was pursued. One patient was discovered to have arrhythmogenic right ventricular dysplasia (ARVD) based on the MRI findings and improved on optimal medical therapy (Table 4).

**Table 1:** Discrepancies between left and right ventricular ejection fraction calculated by both MUGA and FPRNA radionuclide angiography and cardiac MRI demonstrated

Pt	FPRNA RVEF (%)	MRI RVEF (%)	Discrepancy (%)	MUGA LVEF (%)	MRI LVEF (%)	Discrepancy (%)
1	9	23	14	12	12	0
2	56	43	13	15	21	6
3	16	27	11	19	21	2
4	42	25	17	16	13	3
5	32	34	2	22	13	9
6	22	16	6	49	40	9
7	24	32	8	19	21	2
8	44	31	13	24	23	1
9	48	24	24	27	22	5
Mean	32.6	28.3	12	22.6	20.7	4.1

FPRNA tended to over-estimate RVEF. Correlation between RVEFs from CARDIAC MR and FPRNA was 0.5. Correlation between LVEFs from CARDIAC MR and MUGA was 0.9. The degree of discrepancy in RVEF between FPRNA and CARDIAC MR was statistically significant (*p* = 0.006) relative to the degree of discrepancy in LVEF between MUGA and CARDIAC MR

**Table 2:** The agreement between RV functional analysis determined by cardiac MRI was calculated for both echocardiographic tricuspid annular systolic excursion (TAPSE) and fractional area of change (FAC)

Patient	MRI RVEF (%)	Echo TAPSE (mm)	Echo FAC (%)	Days between Echo and MRI Exams
1	23	26	17	120
2	43	20	27	51
3	27	16	21	20
4	25	12	24	1
5	34	15	13	37
6	16	< 8	15	0
7	32	12	16	0
8	31	12	24	12
9	24	< 8	13	125
Kappa (95% CI)	–	0.39 (0.06 - 0.72)	0.64 (0.21 - 1.00)	

While FAC demonstrated a moderate correlation with FAC (Kappa of 0.69) to cardiac MRI, TAPSE (Kappa of 0.39) only demonstrated a fair agreement

TAPSE abnormal values: mild: 1.3 - 1.5 cm; moderate: 1.0 - 1.2 cm; severe: <1.0 cm

FAC abnormal values: mild: 25 - 31%; moderate: 18 - 24%; severe ≤17%

**Table 3:** Right heart catheterization was performed in 7 of the 9 subjects during heart failure evaluation for pre-operative planning for LVAD or heart transplantation

Patient	MRI RVEF (%)	CVP (mmHg)	MPAP (mmHg)	RVSWI – thermodilution (gm-m/m2/beat)	Days between RHC and MRI Exams
1	23	20	44.67	13.92	117
2	43	12	27.33	17.21	49
3	27	–	–	–	–
4	25	9	26.67	14.12	2
5	34	10	41.67	24.29	22
6	16	*8*	*12.67*	*7.97*	0
7	32	–	–	–	–
8	31	25	45.33	12.17	11
9	24	12	37.00	9.64	125

While patients demonstrated abnormal RV function ranging from mild to severe, RHC parameters used to predict postoperative RVF (i.e., central venous pressure (CVP), mean pulmonary pressure (MPAP), and right ventricular stroke work index (RVSWI)) demonstrated abnormal values in 6 of the 7 patients evaluated with RHC. Italicized values are within normal limits

Normal values: CVP = 3 - 8 mmHg, MPAP = < 25 mmHg, RVSWI = 5 - 10 g-m/m2/beat

**Table 4:** Patient diagnoses and clinical follow-up

Pt	Sex	Age	Diagnosis	Follow-up
1	M	60	NICM	Ischemic bowel; patient expired prior to therapy
2	M	51	ICM	LVAD placed for bridge to therapy
3	M	64	ICM	No device placed; lack of social and unable to quit smoking
4	M	66	NICM	Improved on optimal medical therapy
5	F	59	NICM	LVAD place for bridge to therapy
6	M	64	NICM	Diagnosed with ARVD with cardiac MR
7	M	63	ICM	Improved on optimal medical therapy
8	M	28	NICM	Improved on optimal medical therapy
9	M	58	ICM	LVAD for bridge to therapy; definitive therapy with OHT

Nine patients with diagnosed with advanced heart failure, related to ischemic (ICM) or nonischemic cardiomyopathy (NICM) were retrospectively studied. All patients underwent both cardiac MRI and radionuclide angiography to calculate left and right ventricular function. Clinical follow-up is listed

## Discussion

Patients with advanced heart failure being evaluated for LVAD placement require reliable and reproducible evaluation of right ventricular size and function given the high risk of mortality associated with RVF after LVAD placement. Despite evidence that cardiac MRI provides the most accurate assessment of left and right ventricular size and function, it has not been readily available for heart failure patients given that many of these patients have previously undergone placement of ICD. Despite the recent approval of the first conditional ICD by the FDA, current societal guidelines regard all current, conventional ICDs as a relative contraindication to MRI [44, 45] leading to the continued use of radionuclide angiography.

In our study, we compare the benefits of right ventricular evaluation cardiac MRI for “gold standard” calculation of RVEF with the additional functional and volumetric parameters provided by this modality against ejection fraction calculated by radionuclide angiography. While LVEF calculation using MUGA demonstrated an expected strong correlation (R of 0.9) with cardiac MRI, our research demonstrated only a moderate correlation (R of 0.5) between RVEF using FPRNA compared to cardiac MRI. Additionally, the discrepancy between RVEF calculated by cardiac MRI and radionuclide angiography was larger than LVEF values from cardiac MRI and MUGA.

Previous studies have shown that non-invasive echocardiographic assessments of such as FAC and TAPSE show a strong correlation to RVEF calculated with radionuclide angiography [42], and while our small study population was unable to produce strong correlation between radionuclide angiography calculated RVEF and echocardiographic parameters, our findings demonstrated a similar agreement between RV functional assessment performed with cardiac MRI when compared to both FAC and TAPSE assessments of RV function with FAC demonstrating a ‘moderate agreement’ and TAPSE demonstrating a ‘fair’ agreement. The agreement of echocardiographic parameters of RV function, especially FAC, suggest that while echocardiography may not provide a calculated RVEF it may serve as an acceptable substitute for FPRNA, which can routinely be performed as a part of echocardiographic assessment of heart failure. Furthermore, while only 7 patients underwent RHC during heart failure evaluation, RHC parameters of RV function (i.e., CVP, MPAP, and RVSWI) were able to detect RV failure 6 of the 7 cases suggesting a supporting role for RHC.

Since the calculation of ventricular ejection fraction using cardiac MRI has been accepted as the most accurate assessment of ventricular function, our results suggest that the use of an RVEF calculated by FPRNA may not be the optimum method for patient risk stratification prior to LVAD implantation given the high risk of mortality in patients who progress to RVF. Conversely, our results validate that LVEF calculation determined by radionuclide angiography is acceptable for calculation of left ventricular EF given the strong correlation (R of 0.9) when compared to cardiac MRI. However, the inability of MUGA to provide additional functional parameters; e.g., left ventricular end-diastolic and end-systolic volumes, stroke volume, etc., should foster continued research for evaluation of left ventricular functional and volumetric assessment in patients with ICDs.

Given the evidence supporting heart failure severity with increasing RV chamber size and the increased mortality with the development of RVF after LVAD implantation, we suggest that further investigation of cardiac MRI for RVEF assessment is warranted given the high discrepancy and a moderate correlation of RVEFs acquired by gold-standard cardiac MRI and the more widely utilized FPRNA. Also, given the utility of RHC for detecting of RV failure, further studies to categorize the severity of RHF into a mild, moderate, and severe classification system of these parameters may provide further utility for pre-operative assessment of RV function.

While cardiac MRI is still limited by both the availability and the complexities of ICD safe MRI protocols, our data suggests that further investigation for surrogate measurements is warranted. Given echocardiographic and hemodynamic examinations both provide multiparametric right ventricular function analysis, our study suggest further study of these parameter may provide a equivocal or improved RV functional assessment compared to first-pass radionuclide angiography techniques when compared to ‘gold standard’ MRI derived values. Also, further study of RV volumetric assessments utilizing echocardiogram and/or cardiac MRI may also be useful given that studies demonstrated that multiple echocardiographic parameters, including smaller RV chamber size or abnormal RV short-axis to long-axis ratio, have been a significant predictor of post-operative RVF after LVAD placement [46].

Although this study showed statistically significant discrepancy between RVEFs calculated using cardiac MRI compared to FPRNA, our findings are limited since it was a retrospective study with a small sample population due to the inclusion criteria applied to the study, which required a careful and strict screening to ensure safe performance of MRI with ICD. Given the small patient population we attempted to strengthen our finding by comparing additional available parameters of RV functional assessment including echocardiography and RHC. Furthermore, continued research into cardiac MRI derived right ventricle metrics, such as echocardiographic stroke volume index or end diastolic volume index, may provide further insight into proper risk stratification of patients being evaluated for the either a left-sided or biventricular assist device thus minimizing the morbidity and mortality associated with post-LVAD implantation RVF.

## Conclusions

Advanced heart failure patients are increasingly being treated with left ventricular assist device placement; however, definitive evaluation of both left and right ventricular function is required due to the increased mortality rate associated with right heart failure after LVAD placement. Our results suggest that FPRNA only has a moderate correlation with RVEF calculated using cardiac MRI. Furthermore, echocardiographic parameters of RV function including TAPSE and FAC demonstrated a fair and moderate agreement, respectively, compared to cardiac MRI suggesting a possible substitute for FPRNA. With the increasing need for identification of patients at risk for right heart failure, further studies are warranted to find a more accurate estimate of RVEF. Since this study and prior studies comparing radionuclide or echocardiographic RV function analysis to cardiac MRI-derived RVEFs produced at best a moderate agreement/correlation, investigation in how these methodologies attempt to reduce the complicated anatomy of RV into a planar measurement would be helpful. Furthermore, refinement of these planar measurements and improved quality control regarding attention to anatomic boundaries may increase agreement with MRI allowing for a more readily available non-invasive assessment of RV function.

The expected increase in availability of MRI-conditional ICDs suggests that evaluation of advanced heart failure patients with cardiac MRI also should be further investigated as a definitive assessment for both left and right ventricular function prior to left ventricular assist device placement, and that cardiac MRI data may serve as the most accurate standard for RV volume and functional analysis for determination of the optimal pre-operative planning technique for patients undergoing LVAD implantation for bridge to transplant or destination therapy, or for other advanced devices such as a BiVAD or TAH.

## Abbreviations

ARVD: Arrhythmogenic right ventricular dysplasia
BiVAD: Bi-ventricular assist device
CVP: Central venous pressure
EDA: End- diastolic area
ESA: End-systolic areas
FAC: Fractional area of change
FPRNA: First pass radionuclide angiography techniques
ICD: Automatic implantable cardiac devices
ICM: Ischemic cardiomyopathy
LVAD: Left ventricular assist device
LVEF: Left ventricular ejection fraction
MPAP: Mean pulmonary artery pressure
MRI: Magnetic resonance imaging
MUGA: Multigated acquisition
NICM: Non-ischemic cardiomyopathy
RHC: Right heart catheterization
RVEF: Right ventricular ejection fraction
RVF: Right ventricular failure
RVSWI: Right ventricle stroke work index
TAH: Total artificial heart
TAPSE: Tricuspid annular plane systolic excursion
